# Breast Cancer Detection and Classification Empowered With Transfer Learning

**DOI:** 10.3389/fpubh.2022.924432

**Published:** 2022-07-04

**Authors:** Sahar Arooj, Atta-ur-Rahman, Muhammad Zubair, Muhammad Farhan Khan, Khalid Alissa, Muhammad Adnan Khan, Amir Mosavi

**Affiliations:** ^1^Riphah School of Computing and Innovation, Riphah International University Lahore, Lahore, Pakistan; ^2^Department of Computer Science, College of Computer Science and Information Technology (CCSIT), Imam Abdulrahman Bin Faisal University (IAU), Dammam, Saudi Arabia; ^3^Faculty of Computing, Riphah International University Islamabad, Islamabad, Pakistan; ^4^Department of Forensic Sciences, University of Health Sciences, Lahore, Pakistan; ^5^Networks and Communications Department, College of Computer Science and Information Technology, Imam Abdulrahman Bin Faisal University (IAU), Dammam, Saudi Arabia; ^6^Department of Software, Gachon University, Seongnam, South Korea; ^7^John von Neumann Faculty of Informatics, Obuda University, Budapest, Hungary; ^8^Institute of Information Engineering, Automation and Mathematics, Slovak University of Technology in Bratislava, Bratislava, Slovakia; ^9^Faculty of Civil Engineering, TU-Dresden, Dresden, Germany

**Keywords:** breast cancer (BC), deep learning (DL), learning rate (LR), machine learning (ML), transfer learning (TL), convolutional neural network (CNN)

## Abstract

Cancer is a major public health issue in the modern world. Breast cancer is a type of cancer that starts in the breast and spreads to other parts of the body. One of the most common types of cancer that kill women is breast cancer. When cells become uncontrollably large, cancer develops. There are various types of breast cancer. The proposed model discussed benign and malignant breast cancer. In computer-aided diagnosis systems, the identification and classification of breast cancer using histopathology and ultrasound images are critical steps. Investigators have demonstrated the ability to automate the initial level identification and classification of the tumor throughout the last few decades. Breast cancer can be detected early, allowing patients to obtain proper therapy and thereby increase their chances of survival. Deep learning (DL), machine learning (ML), and transfer learning (TL) techniques are used to solve many medical issues. There are several scientific studies in the previous literature on the categorization and identification of cancer tumors using various types of models but with some limitations. However, research is hampered by the lack of a dataset. The proposed methodology is created to help with the automatic identification and diagnosis of breast cancer. Our main contribution is that the proposed model used the transfer learning technique on three datasets, A, B, C, and A2, A2 is the dataset A with two classes. In this study, ultrasound images and histopathology images are used. The model used in this work is a customized CNN-AlexNet, which was trained according to the requirements of the datasets. This is also one of the contributions of this work. The results have shown that the proposed system empowered with transfer learning achieved the highest accuracy than the existing models on datasets A, B, C, and A2.

## Introduction

Medical imaging is a valuable tool for detecting the existence of various medical diseases and analyzing investigational outcomes. The use of biomedical imaging in cancer treatment is crucial. Cancer is a major public health issue in the modern world. According to the World Health Organization (WHO), cancer in 2018 caused 9.6 million deaths, and a probable 10 million deaths were caused by cancer in 2020 (1). Cancer tumors are caused by the uncontrollable growth of cells in the breast. One of the most frequent malignancies in women is breast cancer. BC is estimated to attack more than 8% of women at some point in their life. BC can start in any part of the breast. The majority of BC begins in the lobules or ducts. However, BC can be detected early, allowing patients to obtain proper therapy and so increase their chances of survival.

Imaging technologies such as magnetic resonance imaging (MRI), diagnostic mammography (2) (X-rays), thermography, and ultrasound (sonography) can help analyze and identify breast cancer (3). Ultrasound images are used in this proposed study. Breast cancer is classified as benign and malignant. Benign tumor cells only grow in the breast and do not split throughout the other cells. A malignant tumor is made up of cancerous cells that have the ability to expand uncontrollably, spread to other areas of the body, and infect other tissues. Because cancer cells vary in size, shape, and location, automatically detecting and localizing cancer cells in BC images are a huge difficulty. Machine learning (ML) (4) approaches have found widespread use in a variety of domains, including educational prediction, pattern recognition, image editing, feature reduction, defect diagnosis, face identification, micro-expression recognition, NLP, and medical diagnosis. Its greatest potential has been discovered in the diagnosis of breast cancer (5).

Many researchers have proposed numerous strategies for the automatic classification of cells in breast cancer detection in recent decades (6). By identifying nucleus traits, cancerous cells of breast cancer can be classified as benign and malignant. However, the system's efficiency and accuracy decrease as a result of the complexity of typical machine learning procedures such as pre-processing, segmentation, feature extraction, and others. Traditional ML problems can be solved using the recently developed DL technique. With exceptional feature representation, this technique can perform picture classification and object localization challenges. The transfer learning approach used a natural-image dataset such as ImageNet and then applied a fine-tuning technique to solve this problem. The main benefit of transfer learning is that it improves classification accuracy and speeds up the training process.

First, network parameters were pre-trained using the data and used in the required domain, and then the system restrictions were changed for improved performance. This study used a model for the classification and detection using TL. The proposed model has two components. The first component is training, and the second component is testing. BC classification can be done using a CNN pre-trained such as the ResNet50, VGG 16, VGG 19, and Inception V2 Res Net. In this work, we have done the job of BC classification and detection by using the AlexNet model. AlexNet is a powerful model that can achieve high accuracies on even the most difficult datasets. AlexNet is a leading architecture for any object identification task and classification, and it has a wide range of applications in the artificial intelligence field of computer vision. Some previous studies used the AlexNet, but in this work, we used a customized AlexNet model which has not been used before in previous studies. In the customized AlexNet, the first and last three layers of the architecture are modified, and newly modified layers are the image input layer, fully connected layer, classification layer, and softmax layer, although the remaining layers remain fixed. The customized model has all of the features for image processing that it learned during the process of training. The main goal of this project was to detect and classify breast cancer, reduce training time, increase accuracy, and enhance classification performance.

**Figure 1:**
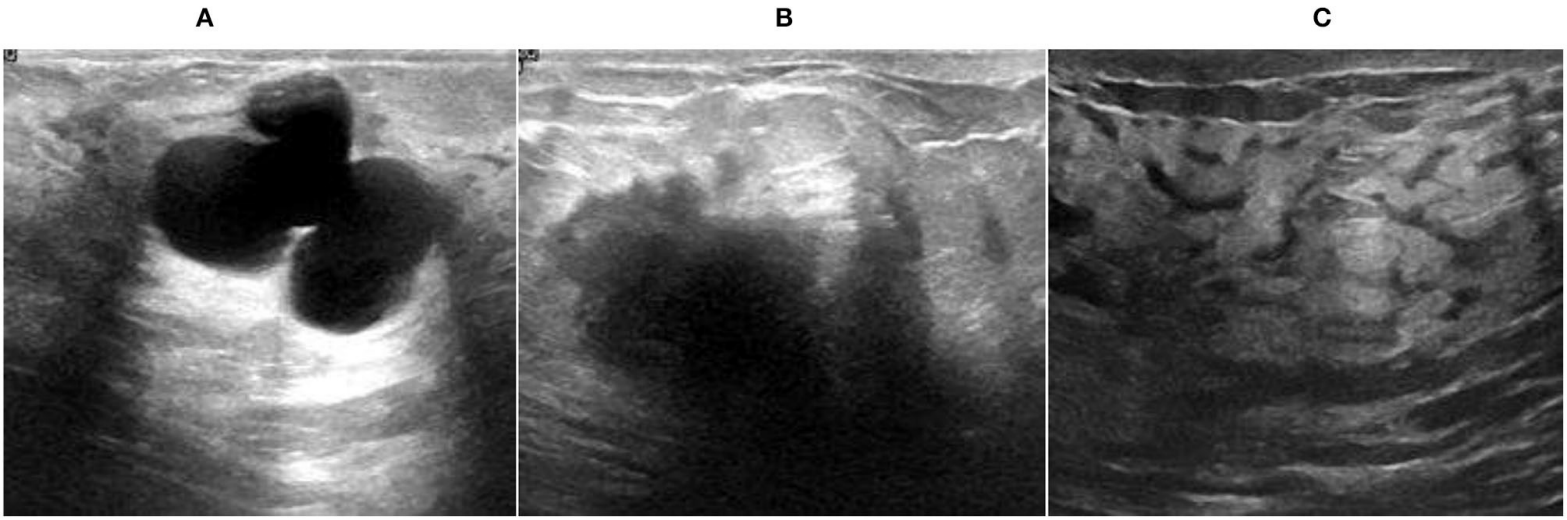
Ultrasound image samples: **(A)** benign, **(B)** malignant, and **(C)** normal.

**Figure 2:**
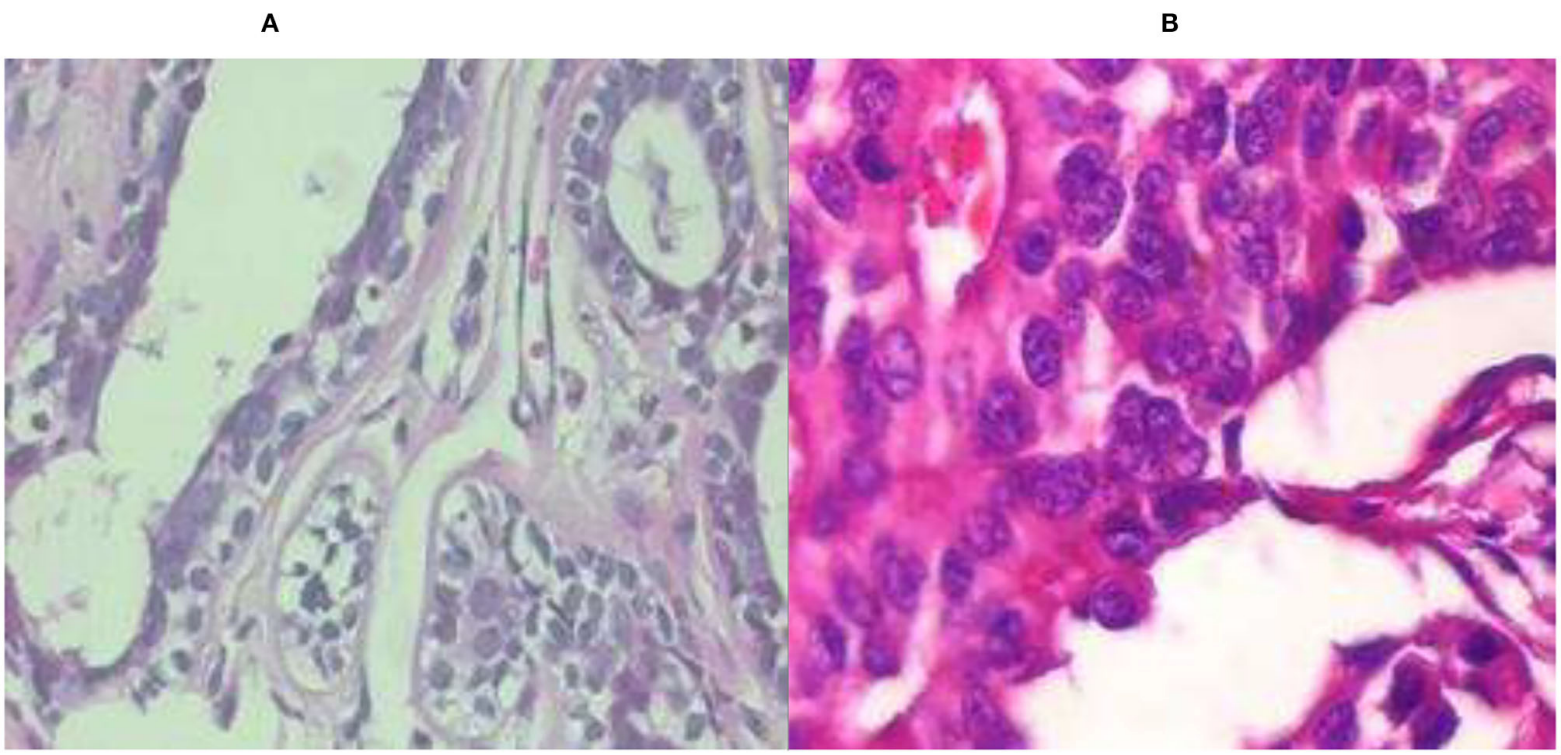
Histopathology image samples: **(A)** benign and **(B)** malignant.

There are many previous studies on breast tumors using various types of models, but with some limitations, breast cancer has limited studies due to the lack of publicly available benchmark datasets. This proposed system worked on three datasets A, B, C, and A2, A2 is dataset A with two classes with a total number of 10,336 images, which is a good dataset. This study is the first to compare three common datasets and suggest the use of customized transfer learning algorithms for breast cancer classification and detection on multiple datasets. By using the customized AlexNet, we achieved the optimum accuracy. This work used ultrasound images and histopathology images, the sample images of ultrasounds are shown in Figure 1, and the sample images of histopathology are shown in Figure 2.

This study is divided into five sections. Section 2 is the literature review, section 3 is the proposed system model, section 4 is the simulation and results, and section 5 is the conclusion of this work.

## Literature Review

Diagnosis of BC disease is a challenge for researchers. To solve this problem of breast cancer, various models and techniques such as ML, DL, and TL are used. Researchers used datasets based on mammography (X-rays), magnetic resonance imaging (MRI), ultrasound (sonography), and thermography to diagnose breast cancer disease.

Fractal dimension (FD) is the best indicator of ruggedness for regular elements, according to their findings. Breast lumps are uneven and can vary from malignant to benign; as a result, the breast is one of the best places to apply fractal geometry. The support vector machine, on the other side, is a new categorization technique. They (2) employed two techniques, FA: SVM and Box Count Method (BCM) in distinct operations that produced good results in respective sectors. The BCM is used to extract features. The retrieved feature “FD” assesses the difficulty of the 42-image input dataset. The generated FD is then processed using the SVM classifier which is used to classify malignant and benign cells. Their highest accuracy is 98.13%.

Breast cancer is a major disease among women between the ages of 59 and 69. They (4) also showed that finding tiny tumors early improves predictions and reduces death rates significantly. Mammography is a useful screening diagnostic method. However, due to tiny changes in tissue densities within mammography pictures, mammography interpretation is challenging. This is particularly true for solid tissues of the breast, and according to this study, screening is more appropriate in greasy breast tissue than in solid breast tissue. Their research focuses on BC detection, as well as danger issues and breast cancer assessments. Their research also focuses on the early diagnosis of BC using 3D MRI mammographic technologies and the classification of mammography pictures using ML.

Their research (5) proposes a heterogeneous efficient machine learning strategy for the early detection of breast cancer. The suggested method follows the CRISP-DM process and employs a stack to construct the collaborative model, which involves three algorithms: KNN, SVM, and decision tree. This meta-classifier's performance is compared to the separate presentations of DT, SVM, and KNN and other particular classifiers NB, SGD, LR, ANN, and a homogeneous collaborative model of (KNN, SVM, DT) and (RF). Using chi-square, the top five characteristics such as glucose, resist in, HOMA, insulin, and BMI are calculated. At K = 20, the proposed collaborative model has the best accurateness of 78% and the smallest log loss of 0.56, denying the null hypothesis. The one-tailed *t*-test, which delivers a lesser consequence at ∞ = 0.05, yields a *P*-value of 0.014.

In this paper (7), they tested the presentation of using conveyed features from a pre-trained model on a dataset of 1,125 breast ultrasound cases. Their dataset is composed of 2,392 regions of interest (ROIs). Each ROI was marked up as cystic, malignant, or benign. Using a convolutional neural network (CNN) (6) from each ROI, features were taken out and used to train (SVM) classifiers. For comparison, classifiers were also trained before retrieving tumor features. CNN-extracted feature-trained classifiers were pretty similar to human-designed feature-trained classifiers. The SVM (8) which was trained on both human-designed features and CNN-extracted features had a 90% accuracy rate in the classification task. The accuracy of the SVM trained on CNN features was 88%, compared to 85% for the SVM trained on features that are human-designed in the task of determining malignant or benign. Deep learning (DL) methods currently in use rely on large datasets. It is worth noting that the study's dataset is not available to the general public.

In this work (8), they look at the potential uses of machine learning for brain problems. They show why machine learning is generating so much interest among researchers and clinicians in the field of brain disorders (9) by highlighting three main applications: predicting sickness onset, assisting with diagnosis, and predicting longitudinal outcomes. They explore the hurdles that must be solved for a successful translational implementation of machine learning in routine psychiatric and neurologic care after exhibiting various applications.

This paper (10) used two datasets of breast ultrasound from two different systems. The first set of data is called breast ultrasound images (BUSI). There is a total of 780 photographs in the BUSI dataset (normal 133, malignant 210, and 437 benign). B dataset has 163 pictures (110 benign and 53 malignant). They used a generative adversarial network (GAN) technology for data augmentation. Researchers can access their BUSI dataset for free. In addition, DL algorithms are applied in this study for breast ultrasound classification. They compare the performance of two alternative methods: a CNN-AlexNet approach and a transfer learning technique with and without augmenting. Their network is trained with a 0.0001 learning rate and 60 epochs. They achieved the accuracies of 94% on BUSI data, 92% on dataset B, and 99% on augmentation.

In this paper (11), they introduce a publicly available collection of 7,909 breast cancer histopathology images. Both benign and malignant images are included in the dataset. The aim connected with this dataset is to automatically classify these photographs into two categories which would be a useful computer-aided diagnosis tool for the clinician. The accuracy ranges from 80 to 85% indicating that there is still space for improvement. In their work to evaluate the feature collection, they used multi-classifiers KNN, SVM, quadratic linear analysis, and RF.

The use of DL techniques for breast ultrasound lesion identification is proposed in this study (12), and three alternative methods are investigated: patch-based Le Net, transfer learning (13), and U-Net approach with the AlexNet model. Two conventional ultrasound picture datasets were obtained, and two separate ultrasound devices are compared and contrasted in this study. Dataset A contains 306 photographs (246 benign and 60 malignant), while dataset B has a total of 163 images (110 benign and 53 malignant). They employed grayscale ultrasound pictures that were divided into 28 × 28 patches. RMS Propagation with LR of 0.01 and 60 epochs is used to train the network. They used the AlexNet model to attain a maximum accuracy which is 91% for dataset A and 89% for dataset B.

Based on two methodologies cross-validation and 80–20, a DL model based on the TL methodology is built in this study (14) to proficiently help in the automatic identification and identification of the breast cancer suspicious area. Deep learning architectures are designed to solve certain problems. Transfer learning applies what one has learned while working on one problem to another. They used six evaluation metrics to assess the proposed model's performance. To train this model, they used a learning rate of 0.01 and 60 epochs. Transfer learning is effective in detecting breast cancer by categorizing mammogram images of the breast with general accuracy, sensitivity, specificity, precision, F-score, and accuracy of 98.96, 97.83, 99.13, 97.35, 97.6.%, and 95%, respectively.

They (15) investigate a quantitative solution to a machine learning problem in this paper. They used transfer learning to train a set of hybrid traditional neural networks based on Azevedo et al. (15) work. Their mission was to tackle BCDR's difficulty in identifying full-image mammograms as malignant or benign. Data collected in this study were used throughout our research to illustrate the regions of the mammograms that the networks were targeting while measuring various performance indicators. They also indicate that some designs perform much better than others depending on the task. According to their findings, the greatest accuracy is 84%.

They (16) demonstrate in their study that the early detection and classification of breast cancer are critical in assisting patients in taking appropriate action. Mammography images, on the contrary, have low sensitivity and efficiency for identifying breast cancer. Furthermore, MRI has a higher sensitivity for detecting breast cancer than mammography. A novel Back Propagation Boosting Recurrent Widening Model (BPBRW) with a Hybrid Krill Herd African Buffalo Optimization (HKH-ABO) method is created in this study to diagnose breast cancer at an earlier stage utilizing breast MRI data. The system is initially trained using MRI breast pictures. Furthermore, the proposed BPBRW with HKH-ABO mechanism distinguishes between benign and malignant breast cancer tumors. Additionally, Python is used to simulate this model. They demonstrate that their model has a 99.6% accuracy rate.

They (17) constructed four distinct predictive models and offered data exploratory techniques (DET) to increase breast cancer detection accuracy in this study. Prior to the models, researchers dug deep into four-layered critical DET, such as feature distribution, correlation, removal, and hyperparameter optimization, to find the most robust feature categorization into malignant and benign classifications. On the WDBC and BCCD datasets, the proposed approaches and classifiers were tested. To evaluate each classifier's efficiency and training time, standard performance metrics such as confusion matrices and K-fold approaches were used. With DET, the models' diagnostic capacity improved, on polynomial SVM achieving 99.3% accuracy, LR 98.06, KNN 97.35, and EC 97.61% accurateness with the WDBC database.

Their (18) goal was to create a hierarchical breast cancer system model that would improve detection accuracy and reduce breast cancer misdiagnosis. To categorize breast cancer tumors and compare their performances, the dataset was subjected to ANN and SVM. The SVM utilizing radial features produced the best accuracy of classification of 91.6%, whereas the ANN obtained 76.6%. As a result, SVM was used to conclude about the importance of breast screening. The second stage involved applying transfer learning to train AlexNet, InceptionV3, and ResNet101. AlexNet scored 81.16%, ResNet101 scored 85.51%, and InceptionV3 scored 91.3 %, according to the data.

They (19) present a framework based on the notion of transfer learning in their research. In addition, a variety of augmentation procedures, including multiple rotation combinations, scale, and shifting, were implemented to prevent a fitting problem and create consistent outcomes by expanding the number of screened mammography pictures. Their proposed solution was tested on the Screening mammography Image Analysis Society (MIAS) database and achieved an accuracy of 89.5% using ResNet50 and 70% utilizing the NASNet-Mobile network. Pre-trained categorization networks are much more efficient and effective, making them more suitable for diagnostic imaging, especially for short training datasets, according to their suggested system.

They (20) used machine learning-based algorithms to help the radiologist read mammography pictures and classify the tumor in an acceptable amount of time in this study. They extracted a number of features from the mammogram's region of interest, which the physician manually labeled. To train and create the suggested structural classification models, these properties are added to a classification engine. They tested the suggested system's accuracy using a dataset that had never been encountered before in the model. As a result, this research discovered that a variety of circumstances can affect the results, which they ignored after investigating. After merging the selection of features optimization approaches, this study advises employing the optimized SVM or Nave Bayes, which provided 100% accuracy.

Their (21) research focuses on employing TL with fine-tuning and on training the CNN with areas derived from the IN breast and MIAS datasets to apply, evaluate, and compare architectures such as AlexNet, Google Net, Vgg19, and Resnet50 to classify breast lesions. They looked at 14 classifiers, each of which corresponded to benign or malignant microcalcifications and masses, as several previous studies have done. With the CNN, they obtained the best results. With an AUC of 99.29%, an F1 score of 91.92%, accuracy of 91.92%, precision of 92.15%, sensitivity of 91.70%, and specificity of 97.66% on a balanced database, Google Net is the better model in a Cad model for breast cancer.

The effectiveness of BC categorization for malignant and benign tumors was evaluated utilizing several machine learning algorithms (k-NN, RF, and SVM) and aggregation methods to calculate the prediction of BC survival by applying 10-fold cross-validation. Their research (22) used a dataset from WDBC that included 23 selected variables evaluated by 569 people, of whom 212 had malignant tumors and 357 had benign tumors. The analysis was done to look at the characteristics of the tumors using the mean, worst values, and standard error. There are 10 properties for each feature. According to the results, AdaBoost has the maximum accuracy for 30 features (98.95%), 10 mean features (98.07%), and 10 worst features (98.77%) with the lowest error rate. To obtain the best accuracy rate, their recommended approaches are categorized using 2-, 3-, and 5-fold cross-validation. When all approaches were compared, AdaBoost ensemble methods had the highest accuracy, with 98.77% for 10-fold cross-validation and 98.41 and 98.24% for 2- and 3-fold cross-validation, respectively. Nonetheless, 5-fold cross-validation revealed that SVM generated the highest accuracy rate of 98.60% with the least error rate.

Breast cancer affects a large number of people all around the world. Mammography is a key advancement in breast cancer detection. It is difficult for doctors to recognize due to its intricate structure. Their (23) research suggests using a CNN to detect cancer cells early. By separating malignant and benign mammography pictures, detection and accuracy can be greatly improved. The Break His ×400 database comes from Kaggle, and the architectures NASNet-Large, DenseNet-201, Big Transfer (M-r101x1x1), and Inception ResNet-V3 perform admirably. M-r101x1x1 has a maximum accuracy of 90% among them. The most important goal of their research is to use selected neural networks to accurately classify breast cancer. This research could help to enhance the systematic diagnosis of early-stage breast cancer.

Despite the fact that there are several scientific studies on the categorization and identification of cancer tumors using various types of models but with some limitations. Breast cancer has limited studies due to the lack of publicly available benchmark datasets. In their work (14), they have used multiple methods such as ResNet50, inception V3, Inception V2 Res Net VGG 19, and VGG 16 but their dataset is too small and they just work on one single dataset and their maximum accuracy is 98.96. In this work (10), they used two different datasets using transfer learning. Datasets are good, but their maximum accuracy is 94% on the BUSI dataset and 92% on dataset B. In this work (12), they also used two different datasets by using CNN multiple models, and they achieved a maximum accuracy with AlexNet, 91% on dataset A and 89% on dataset B. In their work (11), they used a good and large dataset but they also achieved a maximum accuracy is 80–85%. Table 1 shows the comparison of previous studies in terms of accuracy and limitations. Previous studies (4, 5, 7, 10–12, 14) have some limitations like less number of images in the dataset, less accuracy, hand-crafted features required, lack of diverse datasets, no publically available dataset, and an imbalanced number of images in datasets.

**Table 1:** Comparison and limitations of previous studies.

**Publication**	**Model**	**Accuracy**	**Dataset**	**Limitations**
Swain et al. (4)	SVM	98.13%	Private	• Dataset is small
Nanglia et al. (5)	KNN+ SVM + DT	78%	Private	• Less accuracy • Required hand crafted features
Krizhevsky et al. (7)	SVM	88%	Private	• Required hand crafted features • Dataset is not available publically
Dhabyani, et al. (10)	AlexNett+ VGG 16+ Inception+ Res net+ Nasnet	78%, 88%, 85%, 93%, 94%, 80%, 82%, 80%, 90%, 92%	Public	• Less accuracy
Spanhol et al. (11)	SVM	80%	Private	• Less accuracy • Required hand-crafted features
Yap et al. (12)	AlexNet	91%, 89%	Private	• Use of imbalanced dataset • Dataset is small
Saber et al. (14)	Inception v3 + Resnet 50+ VGG 16+ VGG 19	96%, 94%, 96%, 95%	Public	• Dataset is small • Lack of diverse dataset

The following are the primary contributions of this work:.

- This work used three different datasets of breast cancer and compare their results on the same model.
- Improving the accuracy of classification and detection by customizing the model AlexNet.
- Model proposed achieved the maximum accuracy results using transfer learning approaches.

## Proposed Model

**Figure 3:**
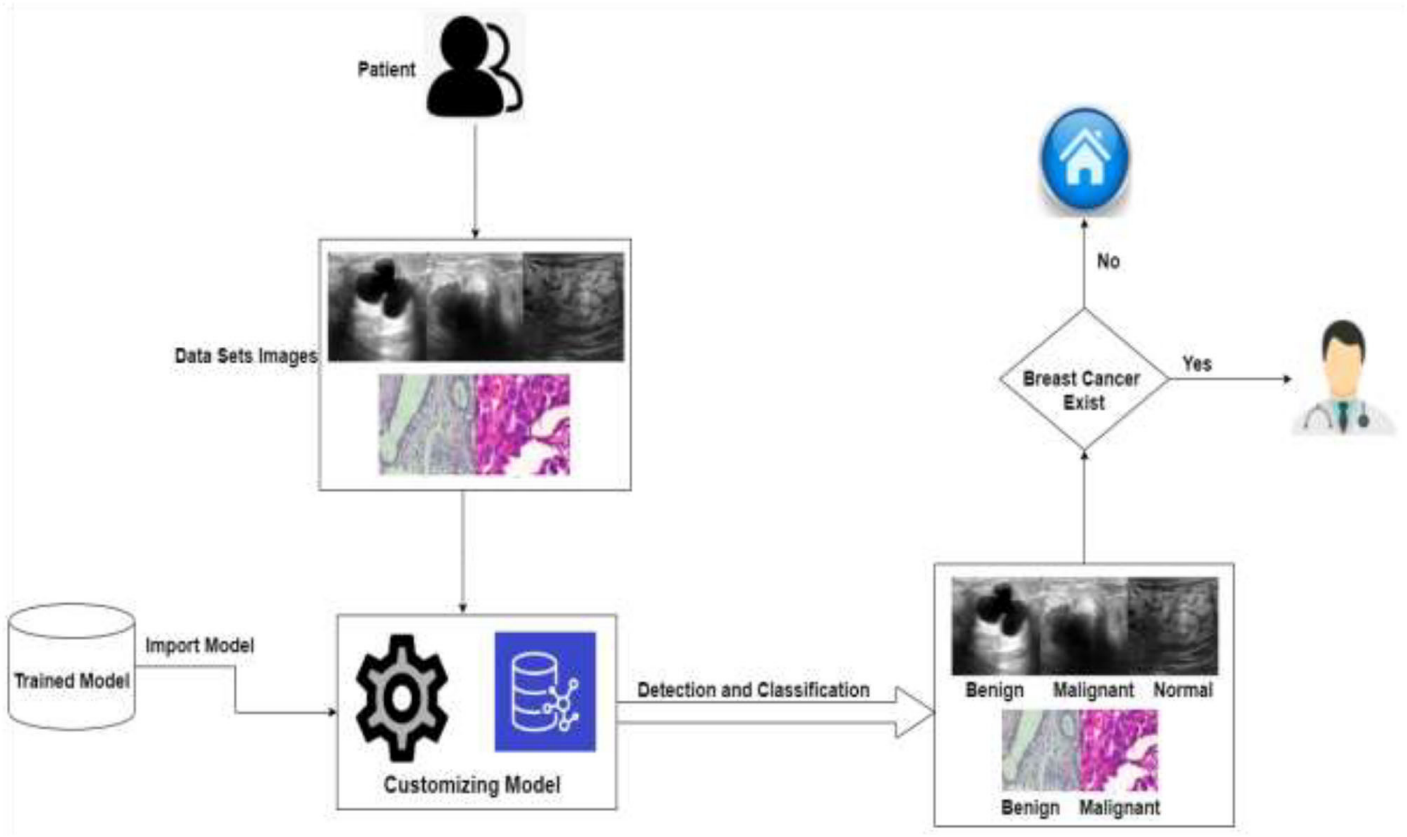
Application level of the proposed system.

In order to assess and identify diseases in medical images, machine learning techniques were applied. Many ML (24) and DL (25) approaches have been widely employed in medical image processing in recent years to detect and evaluate items in medical images. The use of DL techniques to detect breast cancer at an early stage aids medical practitioners in determining its therapy. Breast cancer has been diagnosed early using a variety of DL and transfer learning approaches. DL methods are useful tools for detecting the disease early. Figure 3 shows the application-level representation of the suggested system model.

As we know that in deep learning, few steps are very important: first is data acquisition, data pre-processing, and then the training of the datasets. As we know that if the data are image-based, then the deep learning methods give more accurate results as compared to the machine learning, which is the reason we used deep learning-based solution. There are various kinds of deep learning models like CNN and KNN, as we know that computational resources are also required to compute such kinds of problems like processing power. In further deep learning if we have less computational resources like this one, then we used transfer learning instead of the other deep learning models that is why here we used transfer learning to save the computing power resource optimization. In transfer learning, we used a pre-trained model AlexNet, and we customized this model according to our problem which saves computing power. After that, we stored it in the cloud so that we can use this pre-trained model.

**Figure 4:**
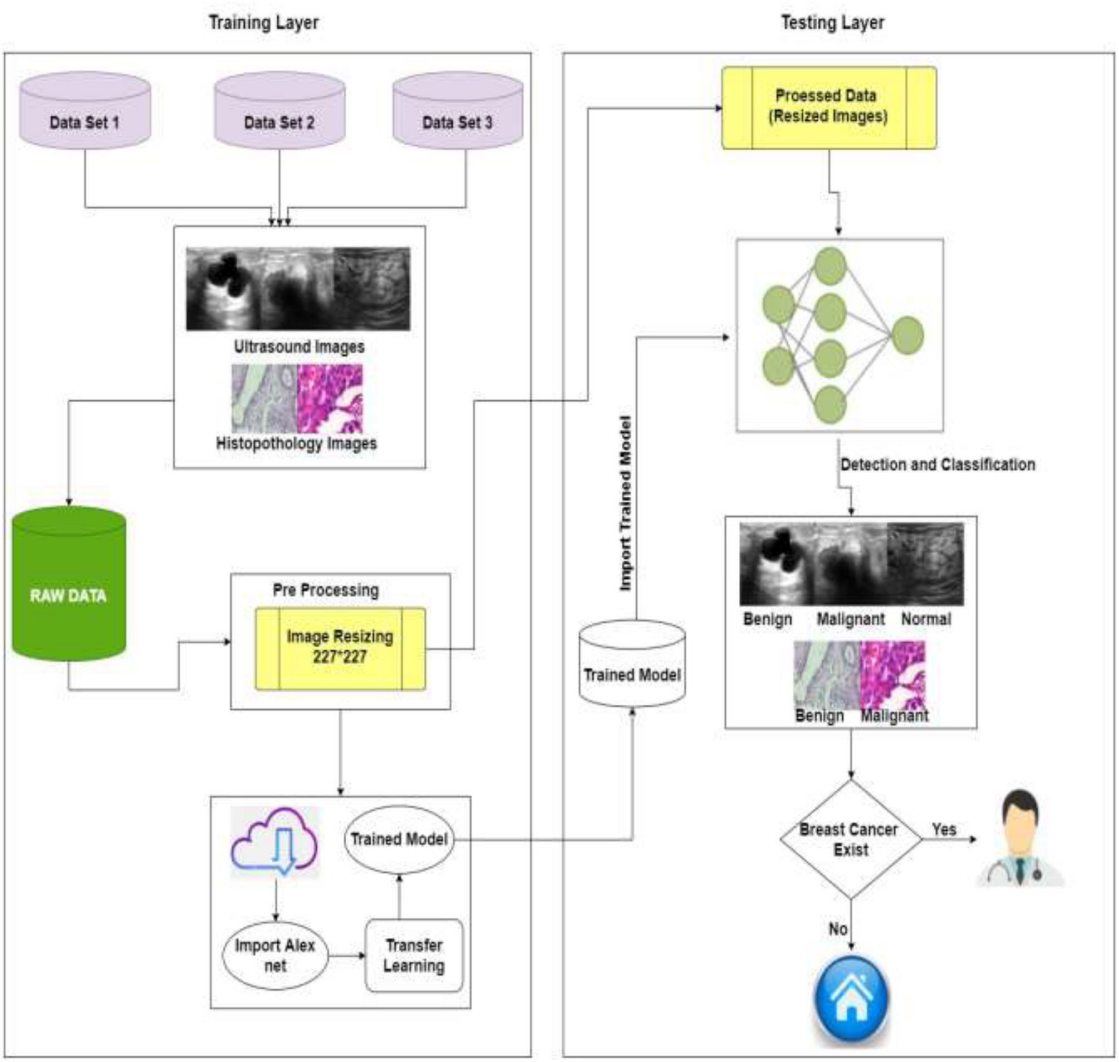
Proposed system model of BC identification and detection.

The detailed proposed system model is shown in Figure 4. The projected method for breast cancer identification and classifications contains two major components. The first component is pre-processing and training, and the second is testing. Based on deep learning techniques, the proposed system model accepts images to help in the classification and early detection of diseases in various stages. Previous research and the Kaggle repository were used to collect the training data, which consisted of ultrasound and histopathology images and the data were collected in raw form. The raw data were handled by the pre-processing layer, which converted the images according to the requirement of the model which is 227^*^227 for AlexNet and customized the pre-trained model AlexNet for transfer learning.

The second layer is training, and for training, this study used pre-processed images of the training layer [227^*^227] and import the customized trained model. The model must be retrained if the learning conditions are not met; otherwise, the trained model is saved in the cloud. The intelligent trained model detects and classifies breast cancer into three categories: benign, malignant, and normal. If the patient is normal, no need to visit the doctor, and if the patient is having symptoms of benign or malignant system, refer her to the doctor for the treatment of BC. Table 2 shows the pseudocode of the proposed algorithm.

**Table 2:** Pseudocode of the proposed model.

1	Start
2	Input breast cancer datasets A, B, C, and A2
3	Pre-processing of the datasets
4	Load data
5	Load pre-trained model
6	Detection and classification of BC using transfer learning model (Customized Alex net)
7	Training phase
8	Store on cloud
9	Image validation phase
10	Compute the performance and accuracy of the proposed model on all dataset by using evaluation matrix
11	Finish

### Dataset

In general, a dataset should be provided to construct a healthcare system employing deep learning. Three separate datasets of breast images are used in this investigation. This study referred to datasets as datasets A, B, and C. Dataset A is collected from (10, 26), dataset B is collected from (11, 27), and dataset C is collected from (28). Dataset A includes medical images of breast cancer obtained by an ultrasound scan. The images in this dataset A are divided into three categories: normal, benign, and malignant. Dataset B contains histopathology pictures of malignant and benign breast cancers, and images were taken as part of a clinical investigation. Dataset C images are also histopathology images. Dataset C is divided into two categories: malignant and benign. We also used dataset A with two classes, benign and malignant, and called it as dataset A2. The number of images in all datasets is shown in Table 3.

**Table 3:** Dataset parameters.

**Data set**	**No. of images**
Dataset A	780 (Benign = 437, Malignant = 210, Normal = 133)
Dataset B	7,783 (Benign = 2,479, Malignant = 5,304)
Dataset C	1,126 (Benign = 547, Malignant = 579)
Dataset A2	647 (Benign = 437, Malignant = 210)

After data collection, pre-processing of images is done. This pre-processing is critical for removing the limitations of abnormality observation and dimension of images according to the AlexNet model. The quality of the images can be increased, and the results can become more precise. Splitting is an important part of a model for training and testing. This proposed model is done by splitting datasets randomly into 80% for the training set and 20% for testing.

### Transfer Learning

Transfer learning is a technique that involves training a CNN model to learn features for a wide range of domains. The proposed TL method is based on AlexNet. The images of the breasts are in grayscale. To make model training easier, pre-processing actions like resizing images into 227^*^227 were taken. This study divided the dataset into training and testing groups randomly, so the models were able to identify significant elements in each image and get a perfect score on the test set. The AlexNet model was used to train all datasets (A, B, and C). The model that has been trained is kept and reused.

This proposed methodology customized the AlexNet model. AlexNet is an eight-layer network with learnable parameters in which three are fully connected layers and five are convolutional layers with max pooling. ReLU is a non-linear initiation function that exists in each layer. Images from the pre-processed layer are read by the network's input layer. The fully connected layers learn disease features to categorize images into specific classes. Early convolutional layers extract common features from pictures by using filters such as detection of edges and preserving the spatial connection between pixels, but later convolutional layers using filters extract general features from images such as detection of edges.

This CNN (29) network was modified to our needs, and the pre-processed images were then loaded into the proposed AlexNet transfer learning model (30). According to the problem, the first and last three layers of the architecture are modified and newly modified layers are the image input layer, fully connected layer, softmax layer, and classification layer, although the remaining layers remain fixed. This customized network is used for TL. The first layer will set the dimension into 227^*^227, and the last three layers are set up according to the labels of the output class and they can categorize the images into their respective groups. The output's size, which is divided into numerous types, is the input parameter for fully connected layers. The fully connected layer in the proposed model will connect three classes: benign, malignant, and normal. Softmax layers are used to apply softmax functions to the input. A fully connected layer learns the class's precise features to differentiate across classes. So, fully connected layers are altered according to dataset classes. To identify images in distinct class labels, this projected network is trained on breast cancer labels of multi-class.

Learning rate and number of epochs are two of the parameters that can be used as training options. The learning rate and epochs are used to train the network. The training was done on various epochs such as 10, 30, and 50, and it was discovered that the ideal epoch was 50, with a learning rate of 0.0008. For training, the stochastic gradient descent with momentum (SGDM) technique of optimization is used. Newly edited layers use these training settings for the breast cancer dataset. The CNN layers are accountable for extracting the general features of images and then for the classification and identification of new datasets by reusing these learning parameters. Models that have been customized and trained are placed on clouds and can be reused. Pre-processed images are passed to the customized model AlexNet during the validation stage. The customized model has all of the features for image processing that it learned during the process of training, so it assesses the images and classifies them into normal, benign, and malignant diseases. After the classification and detection of breast cancer if the patient is normal, no need to visit a doctor, and if the patient has symptoms of disease, then refer to a doctor.

## Simulation and Results

Breast cancer is caused by the uncontrollable growth of cells in the breast. One of the most frequent malignancies in women is breast cancer. BC is estimated to attack more than 8% of women at some point in their life. However, BC can be detected early, allowing patients to obtain proper therapy and so increase their chances of survival. There are many previous studies on breast tumors using various types of models, but with some limitations, breast cancer has limited studies due to the lack of publicly available benchmark datasets. In this study, we worked on three datasets (A, B, C, and A2, A2 is dataset A with two classes) with a total number of 10,336 images which is a good dataset. This study is the first to compare three common datasets and suggests the use of customized transfer learning algorithm AlexNet for breast classification and detection on multiple datasets. Table 14 shows that previous studies do not give accurate results that is why we need a better solution to diagnose breast cancer with more accuracy. Some previous studies used AlexNet, but in this work, we used customized AlexNet model that is not used before in previous studies. The customized model has all of the features for image processing that it learned during the process of training. By using customized AlexNet, we achieved good results that are shown in this section of this study.

In this section, multiple tests were carried out to investigate the performance of this model on datasets A, B, and C. Benign, malignant, and normal were used to categorize the datasets. AlexNet (31) was used to create the proposed model for detecting and classifying breast cancer. The categorization and findings are done in MATLAB 2020a. Evaluation metrics are used to assess produced results. In the training phase, for training, 80% of the dataset is utilized while for testing 20% is used. Transfer learning is applied on AlexNet and compared in form of accuracy (Acc), sensitivity (Sen), specificity (Spe), false-negative ratio (FNR), Miss classification rate (MCR), false-positive ratio (FPR), true positive (TP), false positive (FP), true negative (TN), and false negative (FN) (24, 25). These assessment measures are used to quantify a predictive model's performance.

For binary classes of datasets A2, B, and C.


(1) $$\begin{array}{r} Acc=\frac{C_{P}/G_{P}+C_{N}/G_{N}}{G_{P}+G_{N}}*100 \end{array}$$

(2) $$\begin{array}{r} MCR=\frac{C_{N}/G_{P}+C_{P}/G_{N}}{N}*100 \end{array}$$

(3) $$\begin{array}{r} Sen=\frac{C_{P}/G_{P}}{C_{P}/G_{P}+C_{P}/G_{N}}*100 \end{array}$$

(4) $$\begin{array}{r} Spe=\frac{C_{N}/G_{N}}{C_{N}/G_{N}+C_{N}/G_{P}}*100 \end{array}$$

(5) $$\begin{array}{r} FPR=\frac{C_{N}/G_{P}}{C_{N}/G_{N}+C_{N}/G_{P}}*100 \end{array}$$

(6) $$\begin{array}{r} FNR=\frac{C_{P}/G_{N}}{C_{P}/G_{P}+C_{P}/G_{N}}*100 \end{array}$$

The proposed system classifies datasets A, B, and C into two and three classes, namely benign, malignant, and normal. This work trained datasets on multiple epochs like 10, 30, and 50, and the best accuracy of the proposed model is 99.4% for dataset “A,” 96.7% for dataset B, 99.1% for dataset C, and 100% for dataset A2 on 50 epochs and 0.0008 learning rate. The model proposed for classification and identification of the BC provided improved accuracy as compared to the earlier work of dataset A (10), their accuracy was 94% of dataset B (11), their accuracy was 80% to 85% and no previous work on dataset C, and the proposed model also achieved 100% on dataset A with two classes (dataset A2).

The algorithm is trained on multiple parameters. Transfer learning-based parameters are utilized for training this model and to get the required output in the proposed system. On 10, 30, and 50 epochs to attain optimal accuracy and loss rate, this study trained the model multiple times. Table 4 shows the dataset A classes (benign, malignant, and normal accuracy, respectively, 98.9, 100, and 100% and miss rate, respectively, 1.1%, 0.0%, and 0.0%), dataset B classes (benign and malignant accuracy, respectively, 96.0 and 97.0% and miss rate, respectively, 4.0 and 3.0%), dataset C classes (benign and malignant accuracy, respectively, 99.1 and 99.1% and miss rate, respectively, 0.9 and 0.9%), and dataset A2 classes (benign and malignant accuracy, respectively, 100 and 100% and miss rate, respectively, 0.0 and 0.0%) on 50 epochs.

**Table 4:** Training model on 50 epochs class-wise.

**Data set**	**Classes**	**Epochs**	**Accuracy**	**Miss rate**
A	Benign	50	98.9%	1.1%
	Malignant	50	100%	0.0%
	Normal	50	100%	0.0%
B	Benign	50	96.0%	4.0%
	Malignant	50	97.0%	3.0%
C	Benign	50	99.1%	0.9%
	Malignant	50	99.1%	0.9%
A2	Benign	50	100%	0.0%
	Malignant	50	100%	0.0%

Table 5 shows the accuracy and miss rate of dataset “A” on 10, 30, and 50 epochs. Accuracy is 70.5%, miss rate is 29.5% on 10 epochs, accuracy is 96.8%, miss rate is 3.2% on 30 epochs, and accuracy is 96.8%, miss rate is 3.2% on 50 epochs. Table 6 shows the miss rate and accuracy of dataset B on 10, 30, and 50 epochs. Accuracy is 77.5%, miss rate is 22.5% on 10 epochs, accuracy is 95.6%, miss rate is 4.4% on 30 epochs, and accuracy is 96.7%, miss rate is 3.3% on 50 epochs. Table 7 shows the accuracy and miss rate of dataset C on 10, 30, and 50 epochs. Accuracy is 96.0%, miss rate is 4.0% on 10 epochs, accuracy is 97.3%, miss rate is 2.7% on 30 epochs, and accuracy is 99.1%, miss rate is 0.9% on 50 epochs. Table 8 shows the accuracy and miss rate of dataset A2 on 10, 30, and 50 epochs. Accuracy is 89.1%, miss rate is 10.9% on 10 epochs, accuracy is 96.1%, miss rate is 3.9% on 30 epochs, and accuracy is 100%, miss rate is 0.0% on 50 epochs.

**Table 5:** Training model on dataset A.

	**Epochs**	**Accuracy**	**Miss rate**
Dataset A	10	70.5%	29.5%
	30	96.8%	3.2%
	50	99.4%	0.6%

**Table 6:** Training model on dataset B.

	**Epochs**	**Accuracy**	**Miss rate**
Dataset B	10	77.5%	22.5%
	30	95.6%	4.4%
	50	96.7%	3.3%

**Table 7:** Training model on dataset C.

	**Epochs**	**Accuracy**	**Miss rate**
Dataset C	10	96.0%	4.0%
	30	97.3%	2.7%
	50	99.1%	0.9%

**Table 8:** Training model on dataset A2.

	**Epochs**	**Accuracy**	**Miss rate**
Data set A2	10	89.1%	10.9%
	30	96.1%	3.9%
	50	100%	0.0%

**Figure 5:**
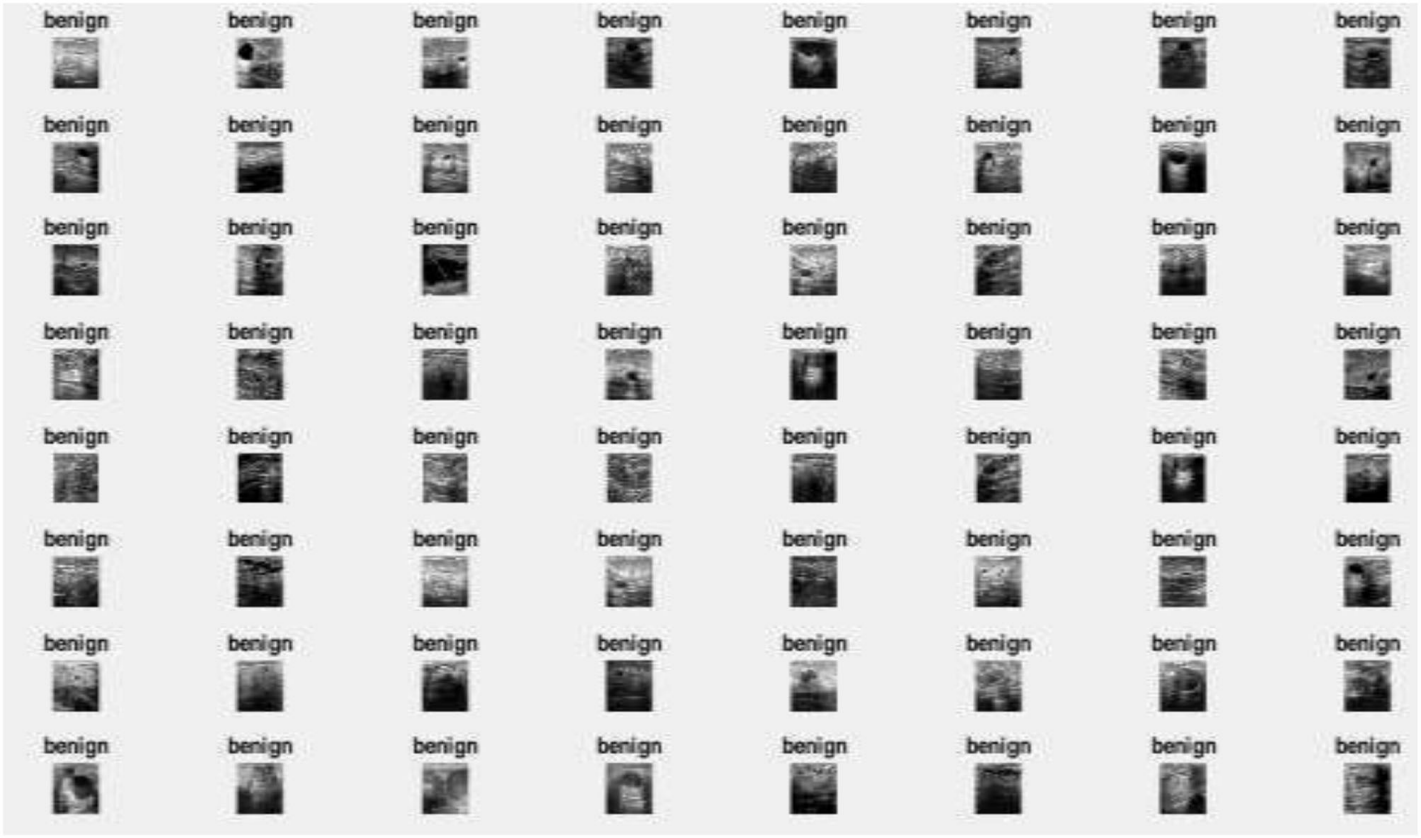
Image classification of dataset A.

**Figure 6:**
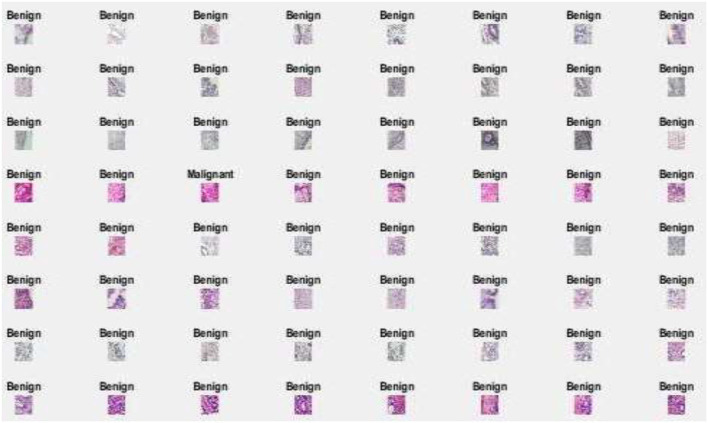
Image classification of dataset B.

**Figure 7:**
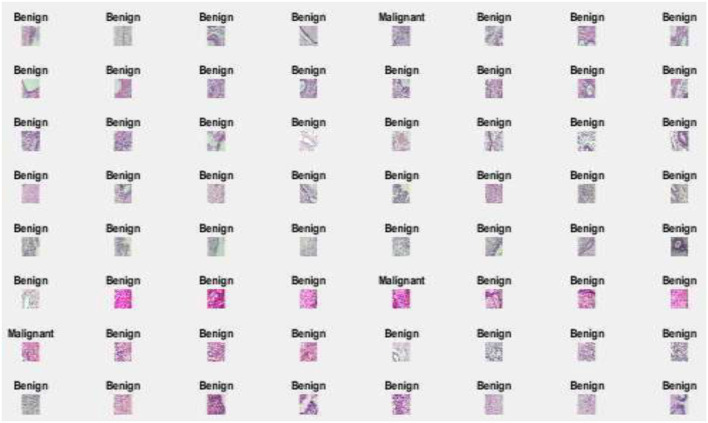
Image classification of dataset C.

**Figure 8:**
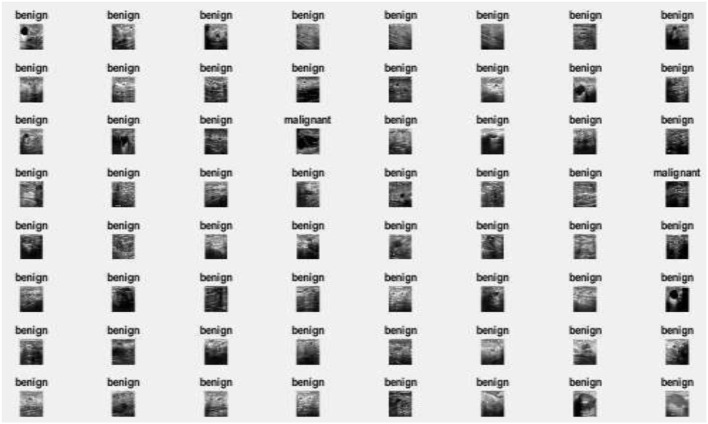
Image classification of dataset A2.

Figure 5 represents the proposed system's labeled pictures of BC according to the dataset “A” classes benign, malignant, and normal. Figure 6 represents according to the dataset B classes benign and malignant. Figure 7 represents according to the dataset C classes benign and malignant. Figure 8 represents according to the dataset A2 classes benign and malignant.

Table 9 shows the confusion matrix of breast cancer classification dataset “A” on 50 epochs. The total number of photographs used for 50 epochs was 780 of dataset “A” with 624 images used for training and 156 images used for testing. A total of 86 images of benign were used for classification in which 86 were classified as benign, 42 images of malignant were used in the classification in which 42 were classified as malignant, and 28 images of normal were used in the classification in which 27 were classified as benign and 1 as benign.

**Table 9:** Confusion matrix of dataset “A” (testing).

**Breast cancer**	**Benign**	**Malignant**	**Normal**
Benign	86	0	0
Malignant	0	42	0
Normal	1	0	27

Table 10 shows the confusion matrix of breast cancer classification dataset B on 50 epochs. The total number of photographs used for 50 epochs was 7,783 of dataset A with 6,226 images used for training and 1,557 images used for testing. A total of 508 images of benign were used for classification in which 476 were classified as benign and 32 as malignant, and 1,049 images of malignant were used for classification in which 1,029 were classified as malignant and 20 as benign.

**Table 10:** Confusion matrix of dataset B (testing).

**Breast cancer**	**Benign**	**Malignant**
Benign	476	32
Malignant	20	1,029

Table 11 shows the confusion matrix of breast cancer classification dataset C on 50 epochs. The total number of photographs used for 50 epochs was 1,126 of dataset C with 225 images used for training and 901 images used for testing. A total of 109 images of benign were used for classification in which 108 were classified as benign and 1 as malignant, and 116 images of malignant were used for classification in which 115 were classified as malignant and 1 as benign.

**Table 11:** Confusion matrix of dataset C (testing).

**Breast cancer**	**Benign**	**Malignant**
Benign	108	1
Malignant	1	115

Table 12 shows the confusion matrix of breast cancer classification dataset A2 on 50 epochs. The total number of photographs used for 50 epochs was 647 of dataset A2 with 518 images used for training and 129 images used for testing. A total of 87 images of benign were used for classification in which 87 were classified as benign and 0 as malignant, and 42 images of malignant were used for classification in which 42 were classified as malignant and 0 as benign.

**Table 12:** Confusion matrix of dataset A2 (testing).

**Breast cancer**	**Benign**	**Malignant**
Benign	87	0
Malignant	0	42

**Figure 9:**
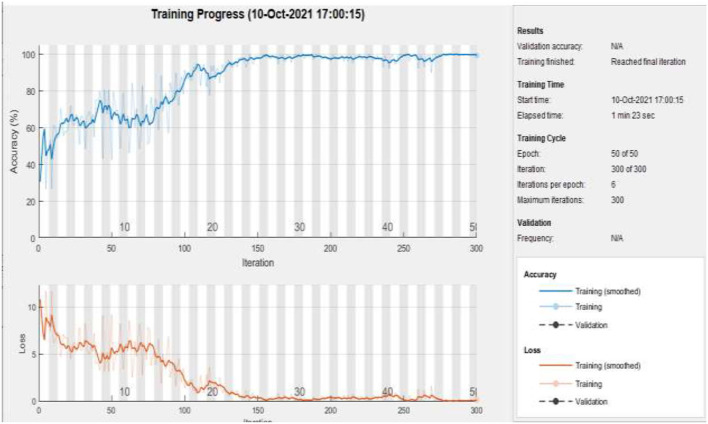
Training of dataset A at 50 epochs.

Figure 9 represents the training accuracy plot which is made up of iterations and epochs and displays the results for 50 epochs of dataset “A.” The precision was initially modest, but as the number of epochs increased, it gradually improved. The proposed system is trained at a learning rate of 0.0008 and a total number of six repetitions for each epoch. The chart depicts the percentage of accuracy for training that began at 1 epoch and ended at 50 epochs.

**Figure 10:**
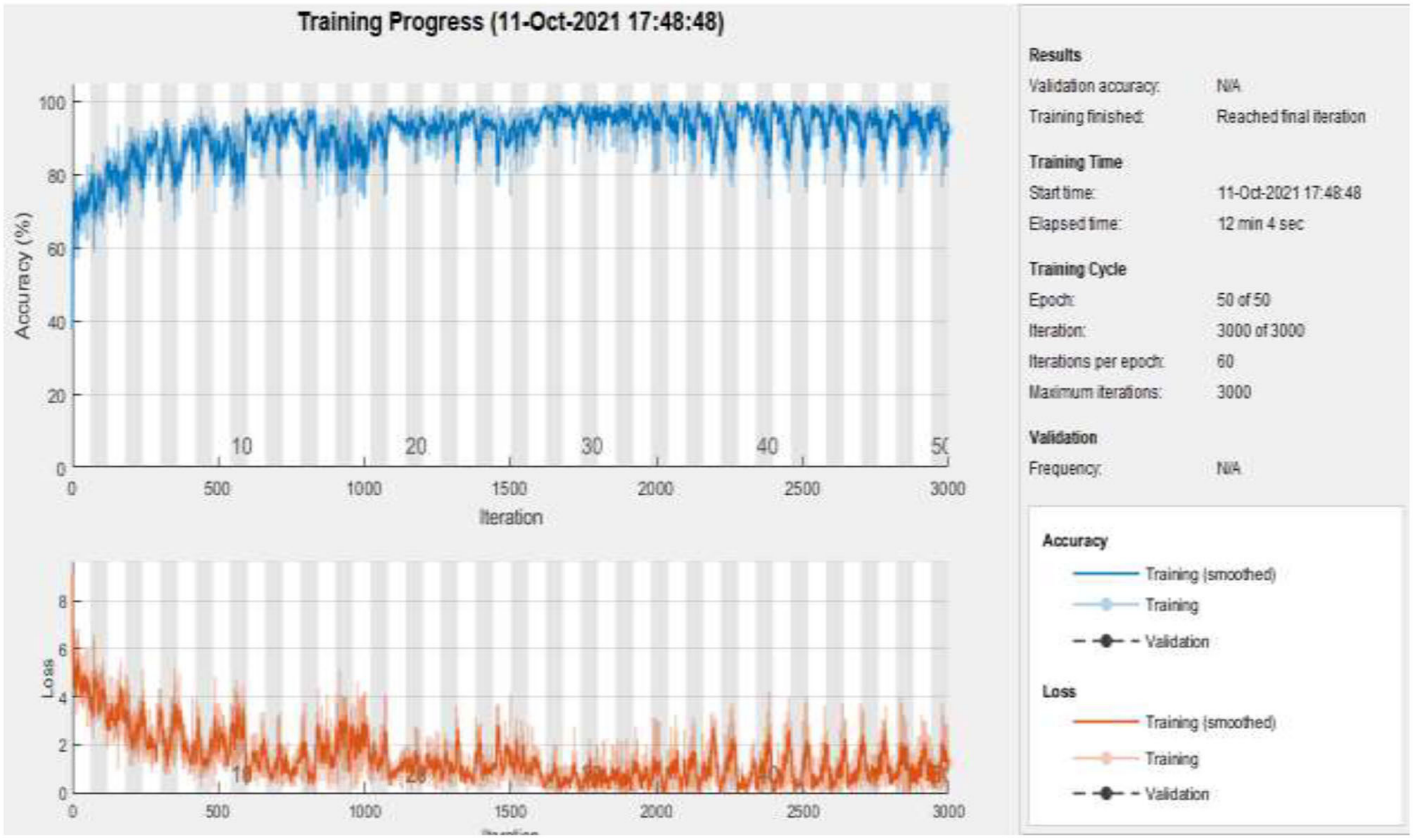
Training of dataset B at 50 epochs.

Figure 10 represents the training accuracy plot which is made up of iterations and epochs and displays the results for 50 epochs of dataset B. The precision was initially modest, but as the number of epochs increased, it gradually improved. The proposed system is trained at a learning rate of 0.0008 and a total number of 60 repetitions for each epoch. The chart depicts the percentage of accuracy for training that began at 1 epoch and ended at 50 epochs.

**Figure 11:**
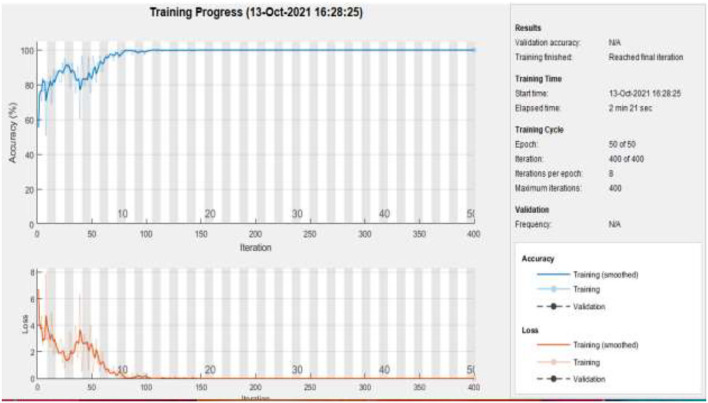
Training of dataset C at 50 epochs.

Figure 11 represents the training accuracy plot which is made up of iterations and epochs and displays the results for 50 epochs of dataset C. The precision was initially modest, but as the number of epochs increased, it gradually improved. The proposed system is trained at a learning rate of 0.0008 and a total number of eight repetitions for each epoch. The chart depicts the percentage of accuracy for training that began at 1 epoch and ended at 50 epochs.

**Figure 12:**
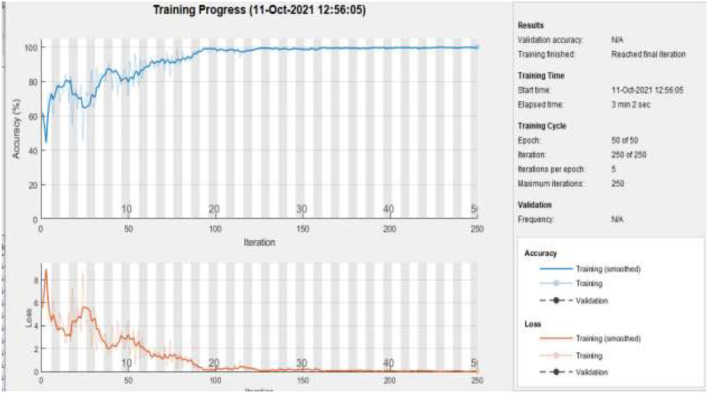
Training of dataset A2 at 50 epochs.

Figure 12 represents the training accuracy plot which is made up of iterations and epochs and displays the results for 50 epochs of dataset A2. The precision was initially modest, but as the number of epochs increased, it gradually improved. The proposed system is trained at a learning rate of 0.0008 and a total number of five repetitions for each epoch. The chart depicts the percentage of accuracy for training that began at 1 epoch and ended at 50 epochs.

The proposed model gives the more precise results as shown in Table 13, and it gives TP of class benign 86 from 87, malignant 42 from 42, and normal 26 from 27. The proposed model gave precise results which are shown in Table 14; on dataset “A,” it gives 99.35% accuracy and 1.149 % FNR for class benign, 100% accuracy and 0.0 % FNR for class malignant, and 99.35% accuracy and 0.0 % FNR for class normal. Table 15 shows that the proposed model gives 96.66% accuracy and 4.03% FNR on dataset B, 99.11% accuracy and 0.9174% FNR on dataset C, and 100% accuracy and 0.0% FNR on dataset A2.

**Table 13:** TP, FP, FN, and TN of dataset A.

**Data set A**	**TP**	**FP**	**FN**	**TN**
Benign	86	0	1	69
Malignant	42	0	0	115
Normal	27	1	0	128

**Table 14:** Statistical measures of dataset A.

**Classes of dataset A**	**Acc**	**MCR**	**Sen**	**Spe**	**FPR**	**FNR**
Benign	99.35%	0.64%	98.85%	100%	0%	1.149%
Malignant	100%	0%	100%	100%	0%	0%
Normal	99.35%	0.64%	100%	99.22%	0.0077%	0%

**Table 15:** Statistical measures of datasets B, C, and A2.

**Data sets**	**Acc**	**MCR**	**Sen**	**Spe**	**FPR**	**FNR**
Dataset B	96.66%	3.339%	95.96%	96.98%	3.016%	4.03%
Dataset C	99.11%	0.8888%	99.082%	99.13%	0.8620%	0.9174%
Dataset A2	100%	0%	100%	100%	0%	0%

**Figure 13:**
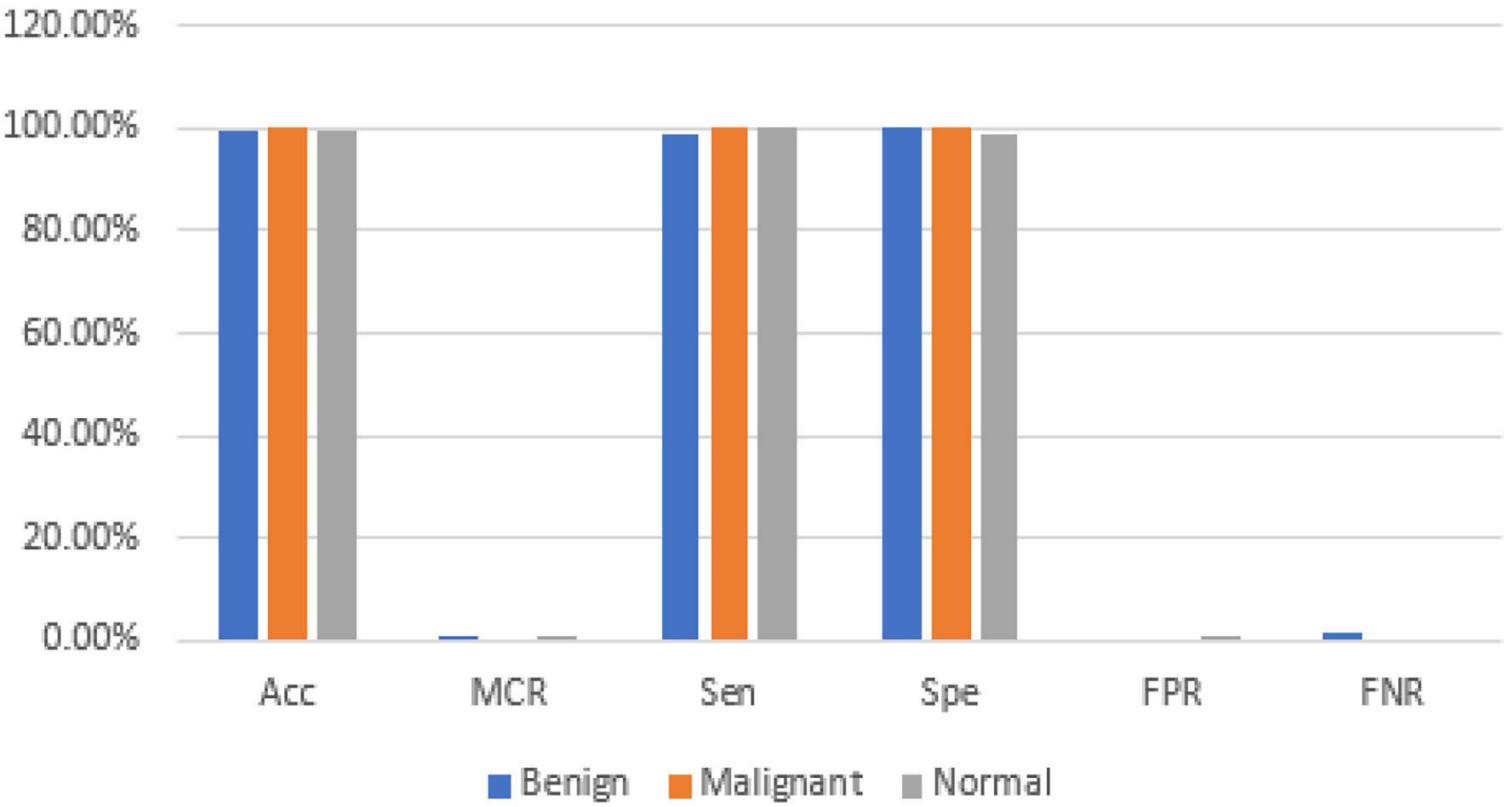
Statistical measures of dataset A.

**Figure 14:**
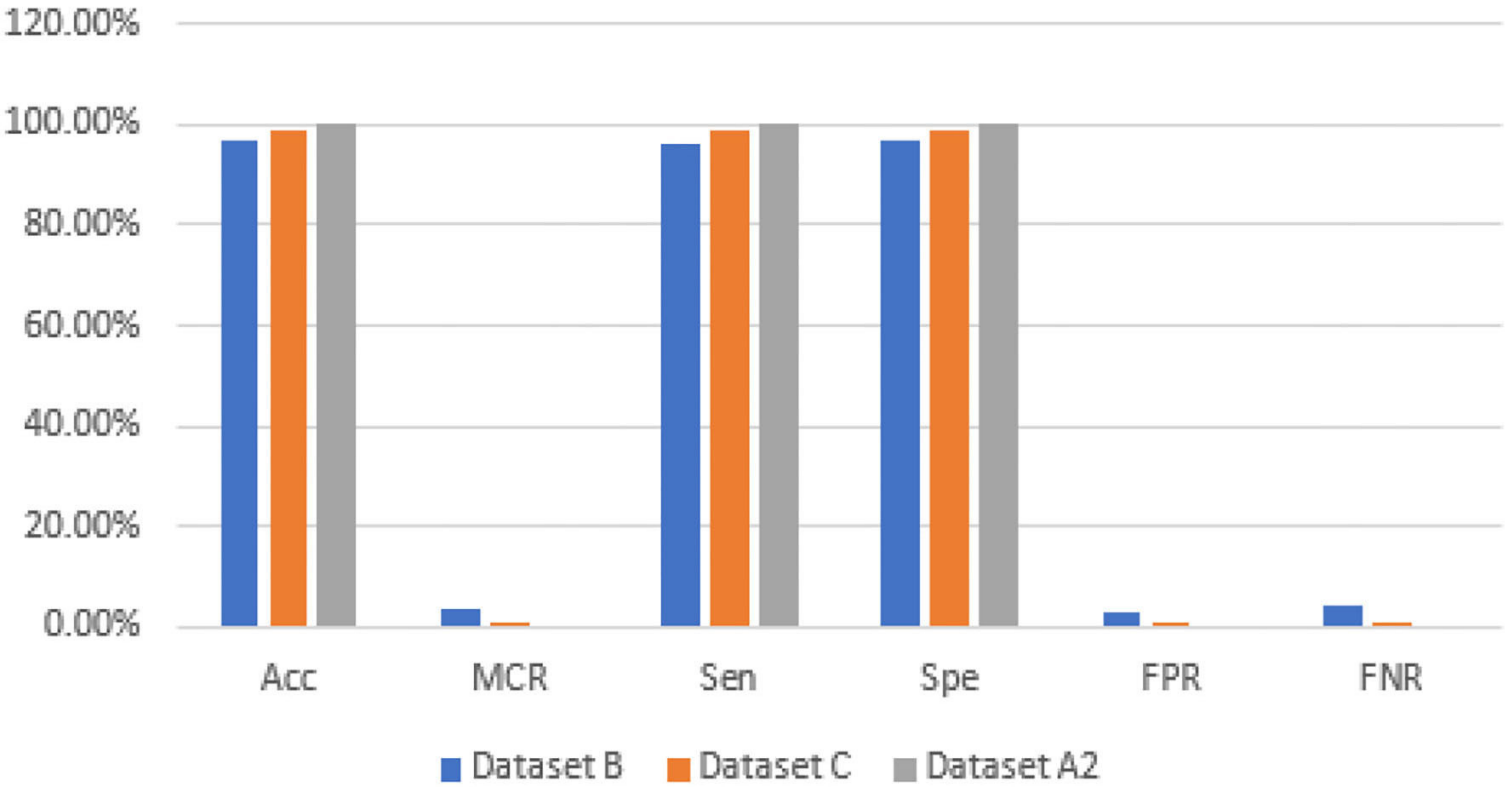
Statistical measures of datasets B, C, and A2.

**Figure 15:**
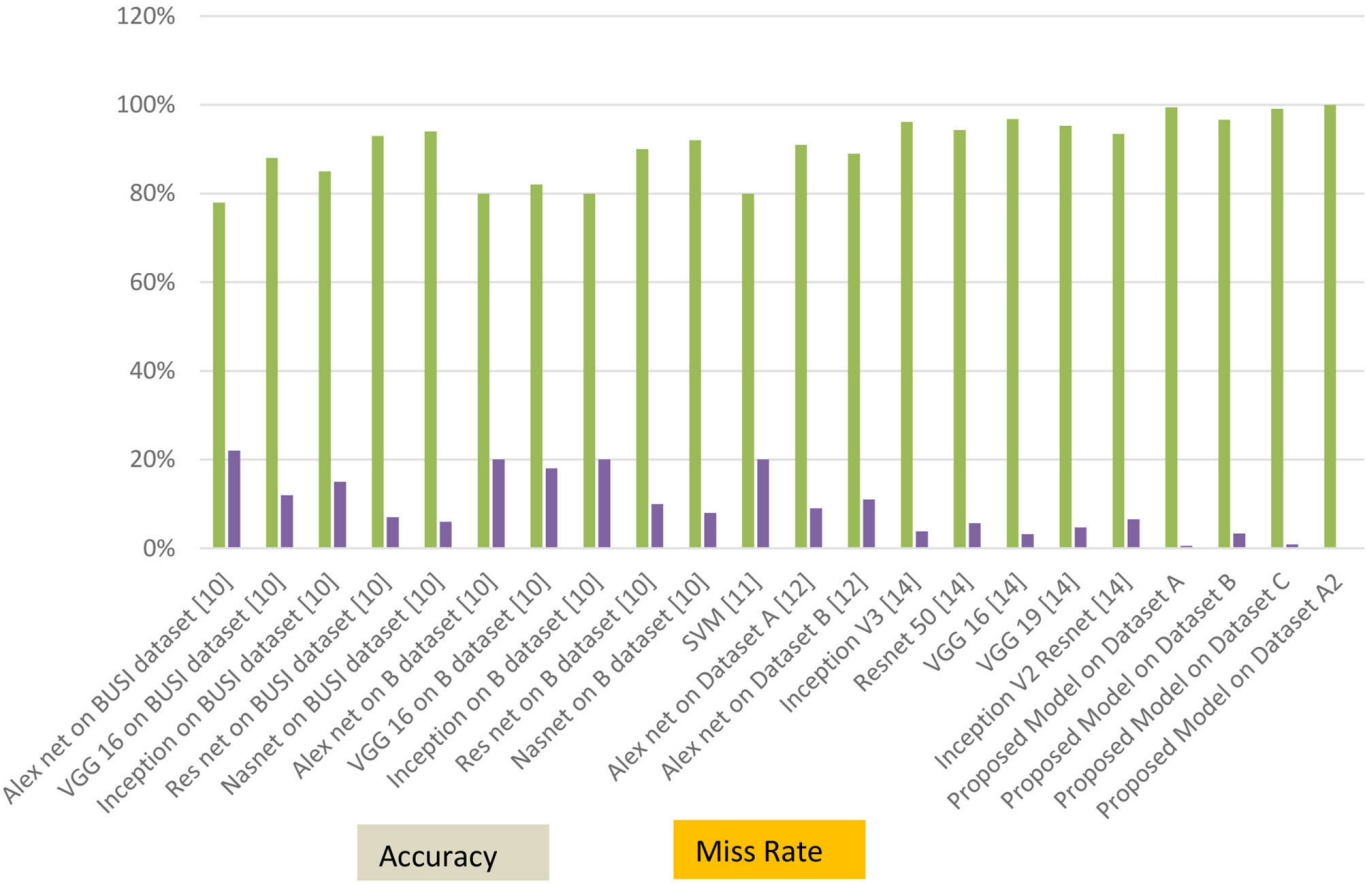
Accuracy and miss rate in contrast with previous studies.

The graphical representations of the statistical measures of dataset “A” are shown in Figure 13, and the graphical representations of the statistical measures of datasets B, C, and A2 are shown in Figures 14, 15. Multiple methods for detecting BC have been utilized in the past. In the identification of a disease for a given class, the proposed methodology attained good accuracy. As a result, early disease diagnosis can assist medical experts in providing treatment to prevent breast cancer spread. Table 15 shows the comparison of the suggested system model's performance with the literature work in terms of accuracy and miss rate. The proposed model obtained an accuracy and miss rate of 99.4% and 0.6%, respectively, on dataset “A,” accuracy and miss rate of 96.66 and 3.34%, respectively, on dataset B, accuracy and miss rate of 99.11 and 0.89%, respectively, on dataset C, and accuracy and miss rate of 100% and 0%, respectively, on dataset A2. These results show that the proposed model achieved accuracy more than the previous models such as AlexNet, VGG 16, Inception, Res net, and NASNet (10) on dataset BUSI and B, SVM (11), AlexNet (12) on dataset A and B, Inception V3, Res net 50, VGG 16, VGG 19, and Inception V2 Res net (14).

## Conclusion

The early detection and classification of breast cancer help to prevent the disease's spread. The use of transfer learning AlexNet on breast cancer classification and detection was examined in this work. Deep learning and transfer learning approaches are adapted to the specific properties of any dataset. The proposed model used the customized AlexNet technique on three datasets, A, B, C, and A2, A2 is the dataset A with two classes. This proposed model empowered with transfer learning achieved the best results by using the customized AlexNet. Dataset A has a maximum accuracy of 99.4%, whereas dataset B has a maximum accuracy of 96.70%, dataset C has a maximum accuracy of 99.10%, and dataset A2 has a maximum accuracy of 100%. In future work, we will apply fusion on these datasets for optimum results. We will also apply other CNN algorithms and our model of machine learning on these datasets.

## Data Availability Statement

The original contributions presented in the study are included in the article/supplementary material, further inquiries can be directed to the corresponding author/s.

## Author Contributions

SA, A-u-R, and MFK have collected data from different resources. SA, MZ, and MAK performed formal analysis and simulation. SA, MFK, KA, and MZ contributed to writing—original draft preparation. A-u-R, MZ, and MAK contributed to writing—review and editing. MAK and AM performed supervision. SA, KA, and MFK drafted pictures and tables. MZ and AM performed revisions and improve the quality of the draft. All authors have read and agreed to the published version of the manuscript.

## Conflict of Interest

The authors declare that the research was conducted in the absence of any commercial or financial relationships that could be construed as a potential conflict of interest.

## Publisher's Note

All claims expressed in this article are solely those of the authors and do not necessarily represent those of their affiliated organizations, or those of the publisher, the editors and the reviewers. Any product that may be evaluated in this article, or claim that may be made by its manufacturer, is not guaranteed or endorsed by the publisher.
